# RETRACTION: Berberine Suppresses EMT in Liver and Gastric Carcinoma Cells through Combination with TGFβR Regulating TGF‐β/Smad Pathway

**DOI:** 10.1155/omcl/9836091

**Published:** 2026-10-27

**Authors:** Oxidative Medicine and Cellular Longevity

RETRACTION: H. Du, J. Gu, Q. Peng, et al. “Berberine Suppresses EMT in Liver and Gastric Carcinoma Cells through Combination with TGFβR Regulating TGF‐β/Smad Pathway,” *Oxidative Medicine and Cellular Longevity*, (2021): 2337818, https://doi.org/10.1155/2021/2337818.

The above article, published online on 18 October 2021 in Wiley Online Library (wileyonlinelibrary.com), has been retracted by agreement between the journal Editor‐in‐Chief, Jeannette Vasquez‐Vivar, and John Wiley & Sons Ltd. The retraction has been agreed due to concerns with the figures. The authors initially raised errors in Figures 4a and 5c, however, on further assessment additional concerns were identified. Specifically:

• In Figure 2a, the HepG2/Scratch/24h 20 and 40 uM similarity is higher than expected
• In Figure 2b, there are unexpected, repeated elements between the HepG2 20 and 40 uM panels after 165 degree rotation
• In Figure 4a, HepG2 ZO1 bands are identical to the MGC803 E‐cadherin bands
• In Figure 5a, the SGC7901 Smad3 bands are identical to the MGC803 Smad3 bands shown in Figure 5c
• In Figure 5c, the HepG2 E‐cadherin bands are identical to the SGC7901 p‐Smad2 bands shown in Figure 5c
• In Figure 5c, the SGC7901 Smad3 bands appear similar to the SGC7901 p‐Smad2 bands shown in Figure 5c when rotated 180 degrees
• In Figure 5c, the HepG2 E‐cadherin bands are identical to the SGC7901 Smad3 bands shown in Figure 5c when rotated 180 degrees
• In Figure 5c, the MGC803 Slug bands appear identical to the MGC803 Slug bands shown in Figure 7b
• In Figure 5c, the SGC7901 p‐Smad3 bands appear identical to the MGC803 p‐Smad3 bands shown in Figure 7b
• In Figure 5c, the MGC803 Vimentin bands appear identical to the MGC803 Vimentin bands shown in Figure 7c
• In Figure 5c, the SGC7901 Smad2 bands appear identical to the MGC803 Smad3 bands shown in Figure 7b
• In Figure 5c, the SGC7901 Smad3 bands appear identical to the MGC803 Smad2 bands shown in Figure 7c

After assessment of the author’s response and the concerns by the editorial board, the results are no longer considered reliable, and the article is retracted.

The authors agree with the retraction.

